# Influence of the Matrix Material and Tribological Contact Type on the Antifriction Properties of Hybrid Reinforced Polyimide-Based Nano- and Microcomposites

**DOI:** 10.3390/polym15153266

**Published:** 2023-07-31

**Authors:** Dmitry G. Buslovich, Sergey V. Panin, Jiangkun Luo, Ksenya N. Pogosyan, Vladislav O. Alexenko, Lyudmila A. Kornienko

**Affiliations:** 1 Laboratory of Nanobioengineering, Institute of Strength Physics and Materials Science of Siberian Branch of Russian Academy of Sciences, 634055 Tomsk, Russia; buslovich@ispms.ru; 2 Laboratory of Mechanics of Polymer Composite Materials, Institute of Strength Physics and Materials Science of Siberian Branch of Russian Academy of Sciences, 634055 Tomsk, Russia; vl.aleksenko@mail.ru (V.O.A.); rosmc@ispms.ru (L.A.K.); 3 Department of Materials Science, Engineering School of Advanced Manufacturing Technologies, National Research Tomsk Polytechnic University, 634050 Tomsk, Russia; jiangkun169@gmail.com (J.L.); knp6@tpu.ru (K.N.P.)

**Keywords:** polyimide, polyetherimide, transfer film (TF), dry sliding friction, wear rate (WR), friction coefficient (CoF), solid lubricant, Raman spectrum, tribological contact, nanoparticles

## Abstract

This paper addresses peculiarities in the formation and adherence of a tribofilm on the wear track surface of antifriction PI- and PEI-based composites, as well as a transfer film (TF) on a steel counterface. It is shown that during hot pressing, PTFE nanoparticles melted and coalesced into micron-sized porous inclusions. In the PEI matrix, their dimensions were much larger (up to 30 µm) compared to those in the PI matrix (up to 6 µm). The phenomenon eliminated their role as effective uniformly distributed nanofillers, and the content of 5 wt.% was not always sufficient for the formation of a tribofilm or a significant decrease in the WR values. At the loaded content, the role of MoS_2_ and graphite (Gr) microparticles was similar, although filling with MoS_2_ microparticles more successfully solved the problem of adhering to a PTFE-containing tribofilm in the point tribological contact. This differed under the linear tribological contact. The higher roughness of the steel counterpart, as well as the larger area of its sliding surface with the same PTFE content in the three-component PI- and PEI-based composites, did not allow for a strong adherence of either the stable PTFE-containing tribofilm on the wear track surface or the TF on the steel counterpart. For the PEI-based composites, the inability to shield the steel counterpart from the more reactive polymer matrix, especially under the conditions of PTFE deficiency, was accompanied by multiple increases in the WR values, which were several times greater than that of neat PEI.

## 1. Introduction

The rapid development of the mechanical engineering, automotive, and aerospace industries increases the requirements for products made from antifriction, wear-resistant materials, including polymer composites [1,2,3]. Among them, polyimide (PI)-based composites are of particular interest due to their high mechanical properties and thermal stability [4,5]. Unfilled PI is rarely used for the manufacturing parts for tribological units because this polymer wears out quickly under dry sliding friction conditions [6,7]. An effective way to solve this issue is to load it with fillers (in the form of fibers and particles), which can serve as both reinforcing inclusions and solid lubricants [8]. Such fillers are represented by carbon [9] and glass fibers [10], PTFE, graphite [11], MoS_2_ [12], and a wide range of nanoparticles of various compositions [13].

Loading with fillers exerts a complex effect on the structure and properties of PI-based composites. Solid lubricant inclusions mainly reduce the strength characteristics because of weak adhesive bonding with the polymer matrix. In this case, an important aspect is the formation of micro- and mesostructures, which is suppressed by the presence of fillers of various natures during the polymer solidification from a melt. Finally, tribological properties are greatly determined by protrusions of filler particles over the friction surface with their subsequent participation in the formation of tribological layers of various thicknesses, structures, and nature [14].

For polymers, the key wear mechanisms are adhesive, strain, and fatigue. Among other things, they are determined by the elastic–viscous behavior of the materials upon tribological loading. The sliding of polymers against solid mating surfaces (mostly metals) can be accomplished with TF formation on a counterface [15], thus determining the development of both friction and wear processes [16]. Under this condition, the contact between the rubbed polymer composite and the metal counterpart might transform from the “polymer-metal” type to the “film-film” type. This phenomenon minimizes the direct contact between the components of a friction pair and reduces the CoF [17], and as a result, reduces its WR.

In addition to fillers of both millimeter and micron lengths, many researchers actively studied the effect of nanoparticles on the friction and wear mechanisms of polymer composites. The main investigated factors are the morphology and chemical composition of a tribofilm on the wear track surface of a composite, as well as a TF on a counterface [18,19,20]. Nowadays, several different types of fillers are loaded more often than single fillers since a possible synergistic effect may greatly contribute to an improvement in the tribological characteristics, thermal stability, and mechanical strength of the composites [21].

For example, [22], Fu et al. investigated the tribological characteristics of PI-based composites loaded with both TiO_2_ nanoparticles and carbon nanotubes (CNTs) under various friction conditions. A sample containing 4 wt.% TiO_2_ and 6 wt.% CNTs showed significantly lower WR and CoF values than those loaded with a single filler. Further investigation of the synergetic effect enabled them to conclude that CNTs enhanced surface hardness, while sheet-like debris reinforced with TiO_2_ acted as a lubricant.

Investigating the tribological characteristics of PI-based composites loaded with nano-SiO_2_ (up to 25 wt.%), Zhao et al. reported [23] that CoF levels decreased by 6.8% when the nano-SiO_2_ content increased from 0 up to 5 wt.%.

In some cases, nanofillers possess complex shapes for improving their adhesion. For instance, in [24], Yuan et al. loaded PI with 0.5–1.5 wt.% MoS_2_ nanoflowers (NFs) by grafting them onto hollow carbon NFs. Such composite coatings showed high wear resistance. With a filler content of 0.5 wt.%, the WR values were lowered by 72.5%.

In [25], Zhou et al. studied PI-based composite coatings filled with CNTs and fluorinated graphene (FG). In these cases, both hybrid and blend phases were mixed with solution doping. Wang Q. et al. reported that the tribological characteristics of PI-based composites were improved by loading with nanoCuO and Gr [26].

In recent papers [26,27] on the tribology of various polymer nanocomposites, the role of nanofillers of different chemical natures was analyzed. It was shown that there was no unique mechanism for the adherence of a tribofilm on the wear track surface of a composite or for a TF on a counterpart. Such processes vary for different test conditions (loading schemes, ambient temperatures, counterface roughness, lubrication conditions, etc.).

In a previous study by [28], the tribological characteristics of both PI- and PEI-based composites reinforced with chopped carbon fibers (CCFs) and filled with nanofillers of various chemical natures (CNTs, HNTs) were investigated in both point and linear tribological contacts. The use of two amorphous high-performance polymers with different chemical structures enabled us to discuss the influence of the flexibility of macromolecules and the activity of the matrix material on the tribological characteristics of the composites. It was shown for both matrix materials that the studied nanofillers did not play the role of a solid lubricant under dry sliding friction conditions (CoF > 0.24). In other words, both PI- and PEI-based studied nanocomposites are not antifriction materials.

To lower both the CoF and WR values, it is necessary to form and adhere an antifriction (tribo)film on the wear track surface of a composite, as well as a TF on a counterpart. Such processes can be promoted by selecting the optimal combination of primary and secondary solid lubricant (micro- and nanosized) fillers, considering their chemical activity [29,30,31].

Under dry sliding friction conditions, the (chemical) activity of the polymer matrix should also be of extreme importance because of two reasons: (i) a possible interaction with the counterpart and (ii) the development of tribochemical reactions between the polymer and, for example, nanofiller particles. Another key role is played by the specific pressure in a tribological contact, the counterpart roughness, and the tribological contact scheme (point, linear, or planar).

Despite the numerous reported data, there is a knowledge gap concerning the influence of solid lubricating nanoparticles on the friction and wear mechanisms of PI- and PEI-based composites, which requires further study. This is of interest in light of the use of widely commercially available solid lubricant particles of nano- and micron dimensions, in particular, PTFE, MoS_2_, and Gr. These fillers perform different functional roles: primary PTFE (chemically inert as most efficient) particles in an amount of 10 wt.% ensures the formation of both tribofilm and TF, while secondary MoS_2_ and Gr particles in a content of 0.5 wt.% provides the possibility for TF adherence to the friction surfaces.

The aim of this paper was to study features of the formation and adherence of a tribofilm on the wear track surfaces of antifriction PI- and PEI-based composites, as well as the adherence of a TF on a steel counterpart. The use of two types of amorphous thermoplastics for manufacturing antifriction composites, on the one hand, is associated with the difference in their chemical structures and, as a result, their chemical activity (PEI is characterized by the presence of “active” oxygen and an ether ring in its molecular chain). On the other hand, the PEI is significantly cheaper and more technologically advanced (easier to process than PI). 

Considering the close mechanical properties of both polymers, PEI is more economical for the design of antifriction composites for metal–polymer friction units. In addition, it is of importance to determine the effect of the polymer matrix material and the tribological contact type on the wear resistance of the PI- and PEI-based composites loaded only with PTFE as well as MoS_2_ or Gr solid lubricant fillers. To compare the efficiency of the micro- and nanofillers in the formation of a tribofilm, both the CoF and WR time-dependent values were analyzed for composites loaded with various combinations of the main (PTFE) and auxiliary (MoS_2_/Gr) micro- and nanofillers. Counterparts from GCr15 bearing steel were used in both the point and line tribological contacts (according to the “ball-on-disk” (B-o-D) and “block-on-ring” (B-o-R) schemes, respectively).

## 2. Materials and Methods

### 2.1. Materials

**Figure 1:**
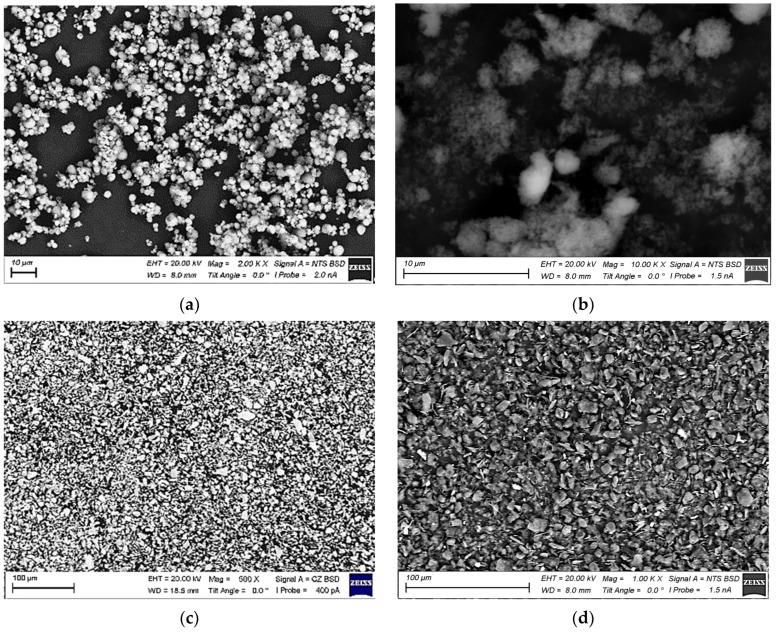
The SEM micrographs of the loaded fillers: µPTFE (**a**), nanoPTFE (**b**), µMoS_2_ (**c**), and µGr (**d**).

The powders used for sample fabrication are listed in Table 1. SEM micrographs (a “LEO EVO 50” scanning electron microscope was used in the backscattering diffraction mode) of the fillers are shown in Figure 1. Note that micron-sized particles were not agglomerated with an even size distribution. In contrast, PTFE nanoparticles were easy to agglomerate in a dry state (Figure 1b) when they were subjected to ultrasonication before loading into the PI/PEI matrix.

### 2.2. Fabrication of the Composites

The details of composite preparation as well as their mechanical and tribological tests were thoroughly reported in our previous papers [11,28]. In order to avoid repetition, this information was moved to the Supplementary Materials.

Similar to earlier studies [32], the PTFE content was chosen to be 10 wt.%; the content of nanoPTFE was 5 wt.%., while the weight fraction of the µMoS_2_ and µGr fillers was equal to 0.5 wt.%. The compositions and designations of the studied composites are presented in Table 2.

## 3. Results

**Figure 2:**
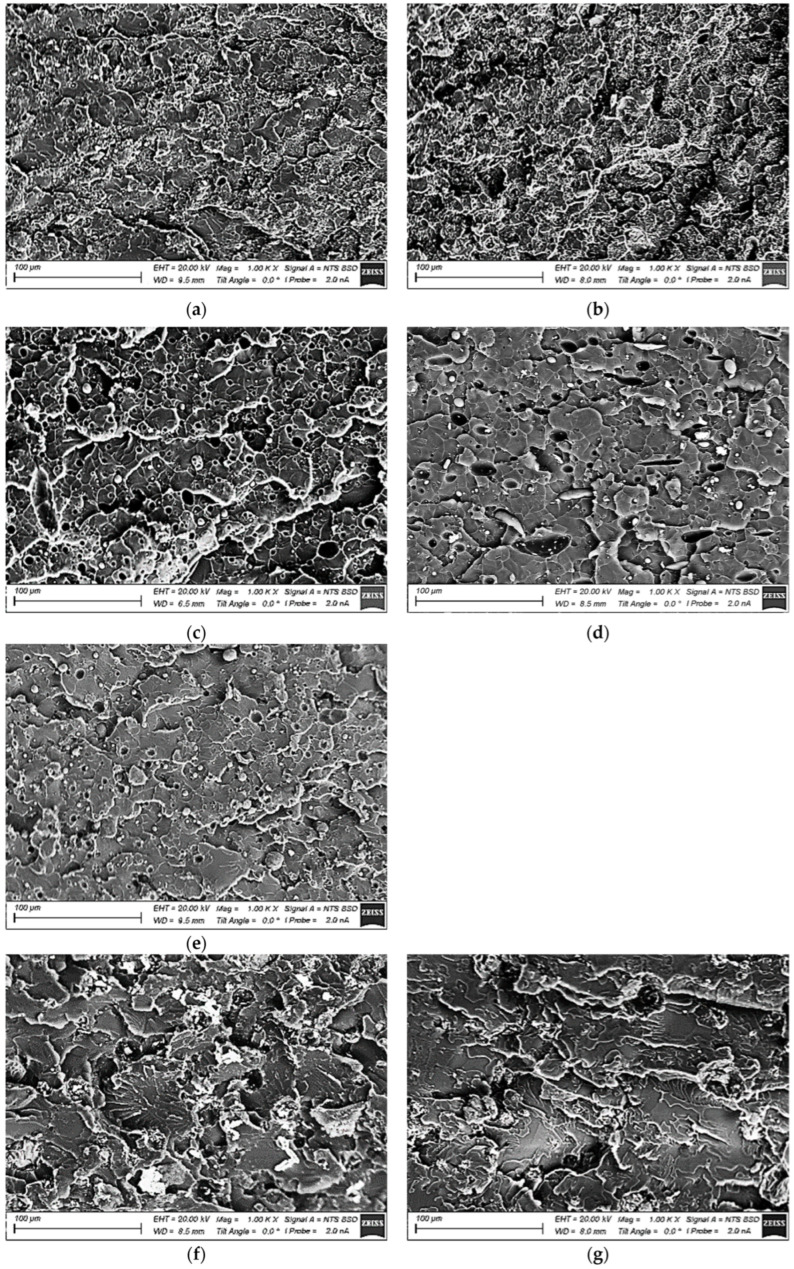

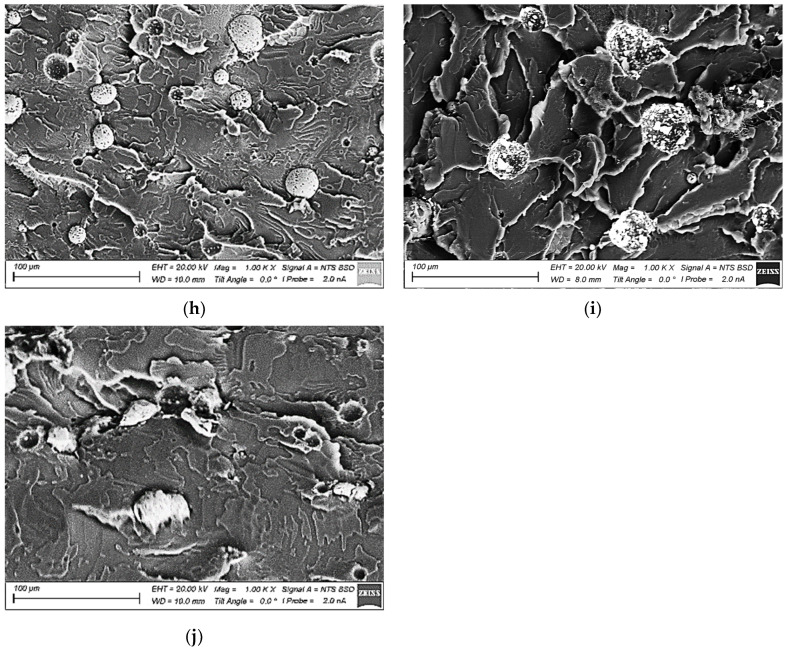
SEM micrographs showing the structure of the PI/10µPTFE/0.5µMoS_2_ (**a**), PI/10µPTFE/0.5µGr (**b**), PI/5nanoPTFE (**c**), PI/5nanoPTFE/0.5µMoS_2_ (**d**), PI/5nanoPTFE/0.5µGr (**e**), PEI/10µPTFE/0.5µMoS_2_ (**f**), PEI/10µPTFE/0.5µGr (**g**), PEI/5nanoPTFE (**h**), PEI/5nanoPTFE/0.5µMoS_2_ (**i**), and PEI/5nanoPTFE/0.5µGr (**j**) composites.

Figure 2 presents SEM micrographs showing the structure of the PI- and PEI-based composites (an “LEO EVO 50” scanning electron microscope was used in the backscattering diffraction mode). SEM micrographs of the loaded fillers are shown in Figure 1. Loading PI with µPTFE particles was accompanied by the formation of a developed microporosity (Figure 2a,b), regardless of the type of auxiliary MoS_2_/Gr inclusions (0.5 wt.%), while filling with 0.5µGr caused a slightly greater heterogeneity (Figure 2b). Loaded nanoPTFE particles fused, coalesced, and formed round inclusions up to 6 µm in diameter (Figure 2c–e). However, no developed porosity, similar to the patterns shown in Figure 2a,b, was found. The addition of 0.5 wt.% of auxiliary µMoS_2_ or µGr, as expected, did not change the character of the formed structure (Figure 2d,e).

In the PEI-based composites, the structure formation proceeded differently. Loading with finely dispersed µPTFE particles was accompanied by the segregation of fluralite inclusions into agglomerates up to a couple of tens of microns in size, regardless of the type of µMoS_2_/µGr inclusions (0.5 wt.%). At the same time, no evident micropores were found within PEI spherulites (Figure 2f,g).

Nevertheless, µPTFE particles did not melt either. The addition of 5 wt.% nanoPTFE particles resulted in their melting followed by coalescence and the formation of large “porous” rounded inclusions up to 30 µm in size (Figure 2h), similar to the PI-based composite. Additional loading with 0.5 wt.% µMoS_2_/µGr did not drastically vary the pattern in the formed structure, but the polymer matrix structure was more uniform (Figure 2i,j). Thus, the PI matrix provided a more uniform distribution of PTFE solid lubricant particles of both micro- and nano-dimensions. Note that µMoS_2_ and µGr were grouped with nanoPTFE fused inclusions; the former was not evident within the polymer matrix.

### 3.1. Mechanical Properties

**Figure 3:**
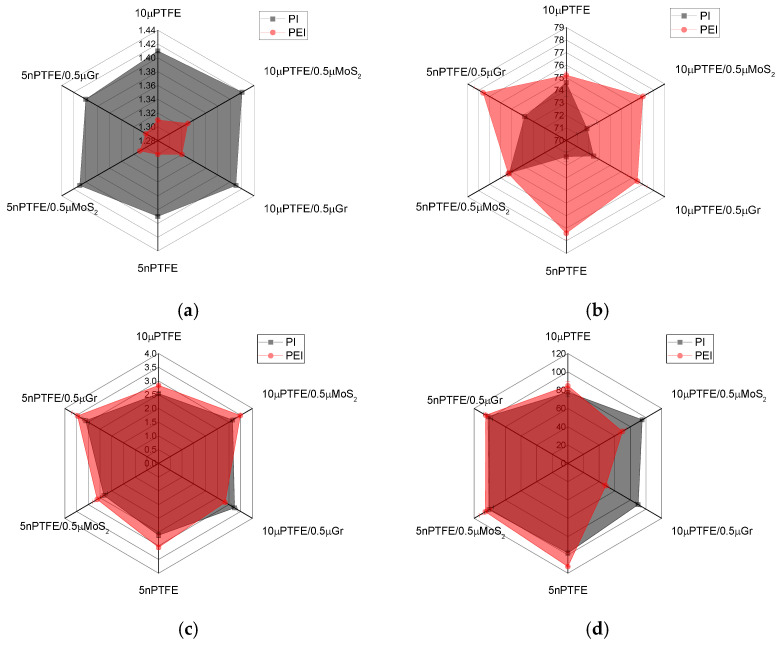
The density (**a**), Shore D hardness (**b**), elastic modulus (**c**), and ultimate tensile strength (**d**) radar diagrams.

The physical and mechanical properties of the composites are presented in Table S1 and Figure 3. First of all, it should be noted that the densities of the PEI-based composites were lower by ~0.1 g/cm^3^ than those of their PI-based analogs. A comparison of the main parameters of the three-component composites loaded with both main PTFE and auxiliary MoS_2_/Gr particles (Figure 3) enabled us to draw the following conclusions:

- The elastic modulus values of the PEI-based composites were higher by 0.3–0.6 GPa than those of the PI-based analogs;
- For the PEI-based composites, the Shore D hardness levels were greater by 4–5 units than those for the PI-based analogs;
- The ultimate tensile strengths of the PI-based composites loaded with µPTFE were higher by 26 and 42 MPa than those for the PEI-based samples.

In general, this result was consistent with the SEM micrographs shown in Figure 2, where the coalescence of PTFE nanoparticles was found in the PEI matrix. At the same time, the UTS of the PI(PEI)-based nanocomposites loaded with PTFE nanoparticles (including those, additionally filled with µMoS_2_/µGr) were close (101–105 MPa). It should be noted that changes in the UTS, as a rule, were proportional to the elongation at break values: the more elongation, the higher the stress at the failure stage.

Thereby, the studied composites differed in both their structures and mechanical properties, depending on the dimensions of the loaded fillers and the polymer matrix.

### 3.2. The Point Tribological Contact

**Figure 4:**
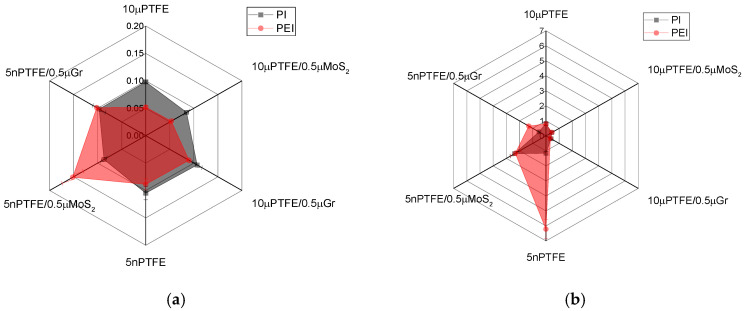
The CoF (**a**) and WR (**b**) radar diagrams; the “B-o-D” scheme.

The mechanical properties of the studied composites differed by a maximum of dozens of percent, while the tribological characteristics varied by many times. Table S2 and Figure 4 present the integral values for the tribological properties obtained in the tribological tests according to the “B-o-D” scheme. Lowering CoF levels down to ~0.1 indicated a correspondence of the composites loaded with PTFE to the “antifriction” status, which was accompanied by multiple decreases in WR values compared to those for neat polymers in the overwhelming majority of cases.

Figure 4 shows the results of the tribological tests. The PEI-based composites predominantly possessed lower CoF levels (Figure 4a), while the WR values were close (with the exception of the PEI/5nanoPTFE sample, Figure 4b).

**Figure 5:**
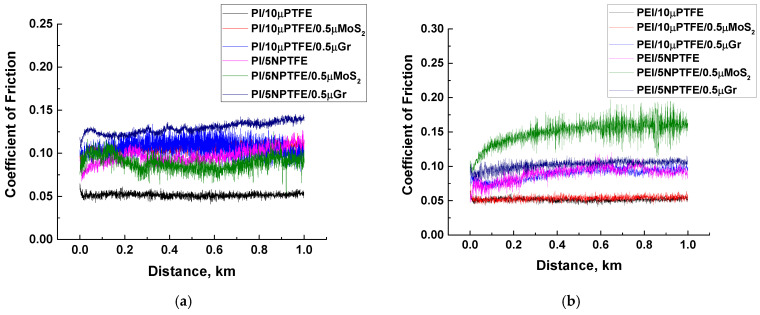
The CoF vs. distance dependencies for the PI- (**a**) and PEI-based (**b**) composites under the “B-o-D” scheme.

The CoF–distance dependencies (Figure 5) reflected the fact that CoF levels were almost constant throughout the tribological tests for all two- and three-component composites, although their average values varied. So, the tribofilm formation mechanism was effective even without loading the polymers with µMoS_2_/µGr. Among the key features, the PEI/5nanoPTFE composite should be noted, which showed a high WR value of 6.22 × 10^−6^ mm^3^/N m despite having a low CoF level of 0.09. To a lesser extent, this manifested itself in the PEI/5nanoPTFE/0.5µGr composite (WR = 1.30 × 10^−6^ mm^3^/N m). In spite of the PTFE content of 5 wt.%, the WR values for the PI-based nanocomposites were close to those for the PI-based composites loaded with 10 wt.% µPTFE.

It is suggested that the high WR values for the PI(PEI)/5nanoPTFE composites could be caused by the low content of solid lubricant particles, which underwent melting and coalescence (Figure 2c,h). So, this could be insufficient for the formation and adherence of a tribofilm (especially in the PEI case). In addition, loading with 10 wt.% nanoPTFE should be recognized as economically inexpedient because 10 wt.% µPTFE (combined with 0.5 wt.% MoS_2_/Gr) effectively improved the antifriction properties.

For the three-component composites, the tribological characteristics were additionally studied at the tripled load of *p* = 15 N (Table 3). This did not change the general trend in the influence of the micro- and nanofillers on the tribological characteristics, despite the WR values being additionally reduced by 1.5 or more times (down to 0.20/0.38 × 10^−6^ mm^3^/N·m, according to Table 3) for the PEI-based composites. For the three-component PI(PEI) nanocomposites, loading with 0.5µGr was more effective: 0.53/0.38 × 10^−6^ mm^3^/N·m versus 1.72/1.78 × 10^−6^ mm^3^/N·m for the PI(PEI)/5nanoPTFE/0.5µMoS_2_, respectively.

**Figure 6:**
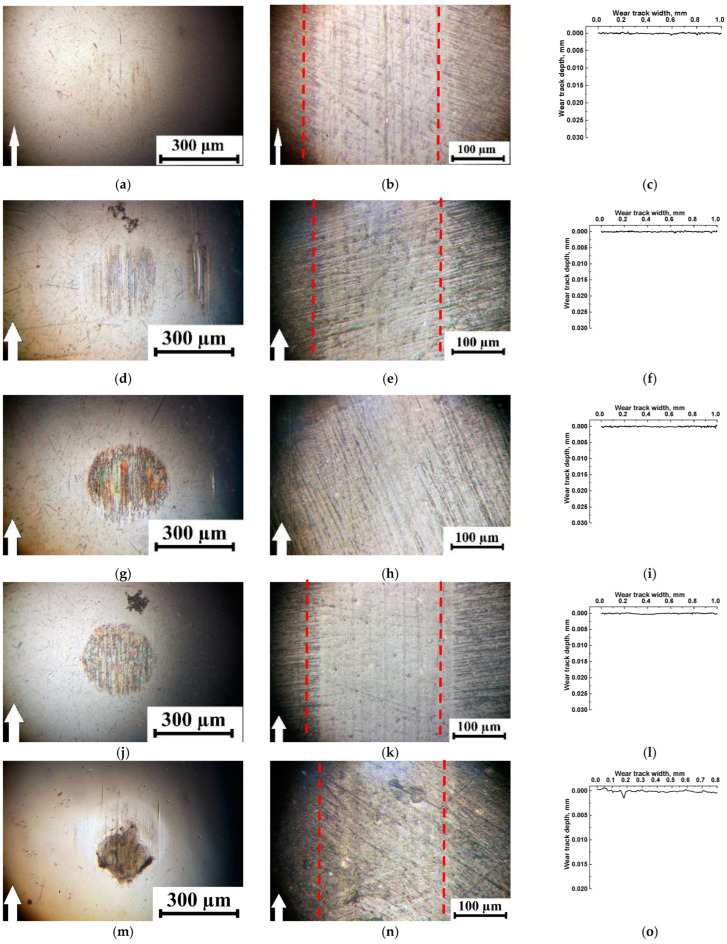

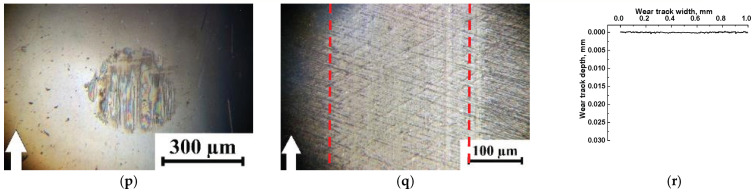
Optical photographs showing the steel counterface (**a**,**d**,**g**,**j**,**m**,**p**) and wear track (**b**,**e**,**h**,**k**,**n**,**q**) surfaces, as well as the wear track profiles (**c**,**f**,**i**,**l**,**o**,**r**) after the tribological tests for the PI/10µPTFE (**a**–**c**), PI/10µPTFE/µMoS_2_ (**d**–**f**), PI/10µPTFE/µGr (**g**–**i**), PI/5nanoPTFE (**j**–**l**), PI/5nanoPTFE/µMoS_2_ (**m**–**o**), and PI/5nanoPTFE/µGr (**p**–**r**) composites under the “B-o-D” scheme; *p* = 5 N, *V* = 0.3 m/s.

Some results for the formation of the tribofilms and TFs were obtained from the optical images shown in Figure 6 for the PI-based composites and the steel counterpart. For the PI/10µPTFE composite, the formation and adherence of a TF on the steel counterface were not practically found, despite a low WR value of 0.8 × 10^−6^ mm^3^/N·m (Figure 6a–c).

All three-component composites were minimally worn (Figure 6f,i,o,r), as evidenced by the remaining scratches from the preliminary surface preparation (Figure 6e,h,n,q). An exception was the two-component PI/5nanoPTFE sample, on which friction against the steel counterpart caused the formation of a pronounced wear track (Figure 6k). At the same time, the formation of a TF of approximately identical size was observed on the steel counterpart surface, which indicated that the tribological contact areas were approximately the same in all cases (Figure 6d,g,j,p). Note that the adherence of irregular shape transfer layer on the steel counterface (Figure 6m) was most likely promoted by the presence of active 0.5µMoS_2_. The latter evidenced higher MoS_2_ activity for TF and tribofilm fixation (at a tribological testing of PI/10µPTFE/µMoS_2_).

We consider that the presence of a TF did not exert a significant effect on the wear intensity in this case. Rather, it indicated that the amount of solid lubricant particles in the tribological contact zone was sufficient to ensure friction at a low CoF level.

**Figure 7:**
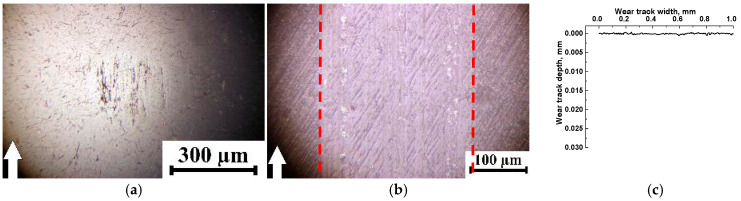

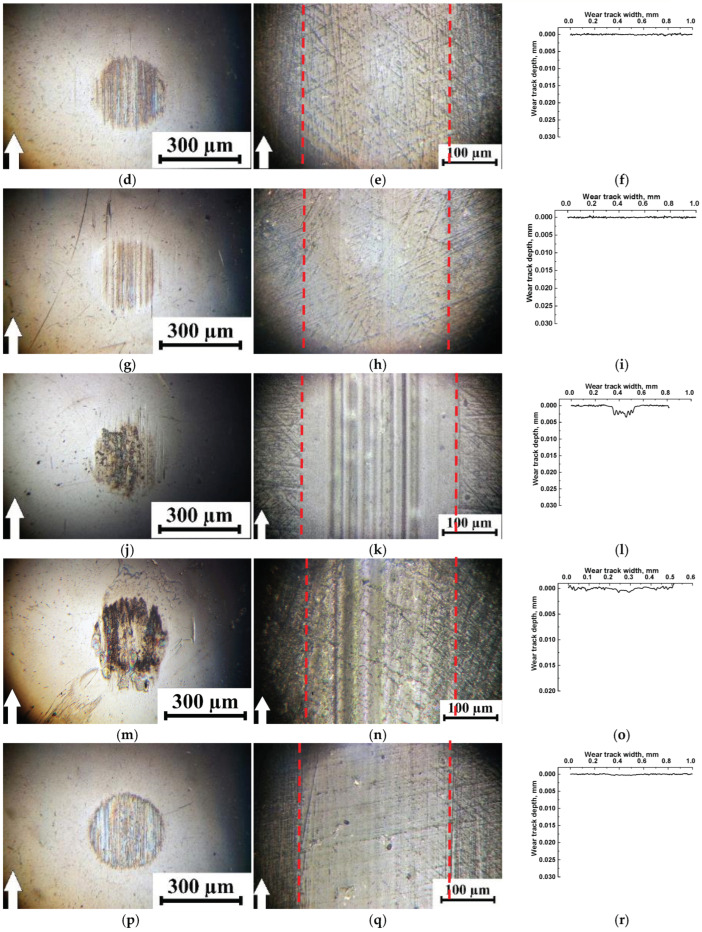
Optical photographs showing the steel counterface (**a**,**d**,**g**,**j**,**m**,**p**) and wear track (**b**,**e**,**h**,**k**,**n**,**q**) surfaces and the wear track profiles (**c**,**f**,**i**,**l**,**o**,**r**) after tribological tests of the PEI/10PTFE (**a**–**c**), PEI/10µPTFE/µMoS_2_ (**d**–**f**), PEI/10µPTFE/µGr (**g**–**i**), PI/5nanoPTFE (**j**–**l**), PEI/5nanoPTFE/µMoS_2_ (**m**–**o**), and PEI/5nanoPTFE/µGr (**p**–**r**) composites under the “B-o-D” scheme; *p* = 5 N, *V* = 0.3 m/s.

Figure 7 shows optical micrographs of the wear track surfaces on the PEI-based composites and the steel counterpart. For the two-component PEI/10µPTFE composite, the formation and adherence of a TF on the steel counterpart did not practically take place (Figure 7a) similar to its PI-based analog with the identical WR value (Figure 7b,c).

In general, the appearance of the wear track surfaces was similar to that on the PI-based composites. Nevertheless, loading with nanoPTFE increased the wear track depths on the two- and three-component composites (Figure 7k,l,n,o,q,r). At the same time, the TF area on the steel counterpart did not increase (Figure 7j,m,p). This could also indicate that the presence of a TF exerted a minimal effect on the wear pattern and, in this case, was rather just a trace reflecting the tribological contact boundaries. In the PEI/5nanoPTFE two-component composite, a TF was characterized by a dark shade and an uneven shape, which could be associated with the development of both tribooxidation and degradation processes, enabling it to attach to the steel counterpart surface (Figure 7j). The adherence of an irregular shape transfer layer on the steel counterface (Figure 7m) was also characteristic of PEI/10µPTFE/0.5µMoS_2_, which was also characteristic of the similarly loaded PI-based composite.

### 3.3. Linear Tribological Contact

Under the “B-o-R” scheme, in addition to the steel counterpart roughness (Ra = 0.2 µm), another important parameter of the tribological tests increased, namely, the contact area. It rose by many times and became “renewable” (i.e., requiring more PTFE to form a TF around the entire perimeter of the steel ring). On the other hand, the wear track, which “supplied” the tribological contact zone with solid lubricant particles, contained a limited number of lubricant particles (determined by the total content of such filler in a composite). In this regard, the fact of the formation and adherence of the tribofilm on the wear track surface of the composite, as well as the TF on the counterpart, was more critical. For this reason, both polymer matrix and counterface materials could exert a more significant effect on the tribological characteristics.

**Figure 8:**
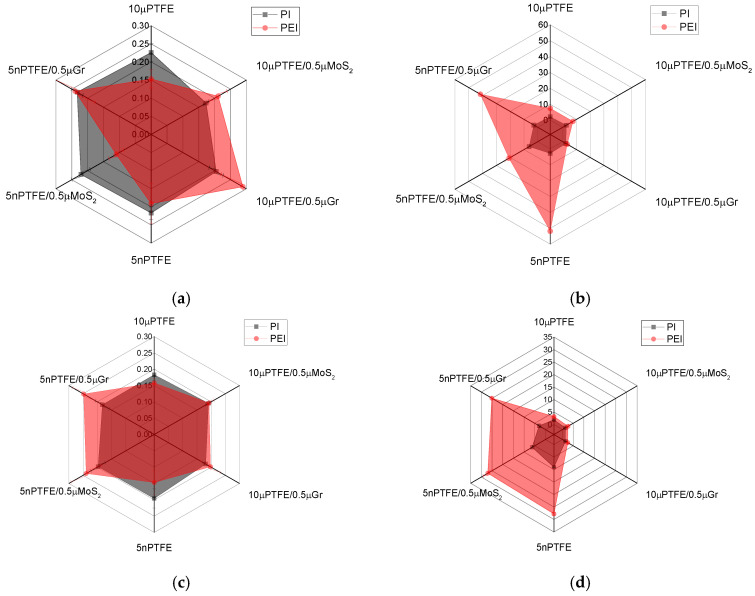
CoF (**a**,**c**) and WR (**b**,**d**) radar diagrams under the “B-o-R” scheme, *p* = 60 (**a**,**b**) and 180 N (**c**,**d**).

According to Figure 8 and Table S3, the mean CoF values were 1.5 times higher for both types of matrix materials compared to those for the point tribological contact (Table S1). This result was clearly associated with both the increase in its area and the higher steel counterpart *R_a_* roughness (0.20 vs. 0.02 µm under the “B-o-D” scheme).

Since the purpose of the loading with 0.5 wt.% MoS_2_/Gr was to adhere a TF, it was expected that the WR values would decrease, in particular, for the ternary PI(PEI)/10µPTFE/0.5µMoS_2_ and PI(PEI)/10µPTFE/0.5µGr composites. However, this fact depended on the matrix material: at comparable CoF levels, the WR values were higher for the two- and three-component PEI-based composites (Figure 8). Multiple increases were observed for the nanocomposites as well (at *p* = 60 N, WR = 51.8 and 41.6 × 10^–6^ mm^3^/N·m for PEI/5nanoPTFE and PEI/5nanoPTFE/0.5µGr, respectively). These values were several times greater and were even greater than those for neat PEI.

In general, the trend could be characterized by changes in the CoF levels as follows (Figure 8). Regardless of the filler load, the average CoF values were about 0.2, with a minimum of 0.110 for the PEI/5nanoPTFE/0.5µMoS_2_ composite (*p* = 60 N) and a maximum of 0.289 for the PEI/10µPTFE/0.5µGr composite (*p* = 60 N). Thereby, the presence of auxiliary fillers MoS_2_/Gr exerted a fundamental effect on the CoF = 0.11 reduction only in one case (for the PEI/5nanoPTFE/0.5µMoS_2_); however, this was accompanied with a multiple wear intensity enlargement WR = 20.9 × 10^−6^ mm^3^/N·m. In contrast, the other PEI-based nanocomposites were many times inferior to their PI-based analogs in terms of their WR values. For the three-component PI-based composites, the WR values were lower by 1.5–3.5 times than those for the identically loaded PEI-based composites.

On the one hand, the decrease in CoF levels for the composites loaded with PTFE compared to neat polymers indicated that the presence of µPTFE facilitated friction. So, the composites approached the “antifriction” status to a certain extent. Nevertheless, the question regarding the mechanism underlying the formation and fixation of a tribofilm on the wear track surface remained open.

In this way, it was shown that the CoF reduction did not always correlate with the decrease in the WR values in the linear tribological contact. In addition, loading PI with nanoPTFE was not accompanied by a noticeable decrease in the WR value, while it led to multiple increases in this parameter for the PEI-based nanocomposites. This fact was quite consistent in the SEM micrographs (Figure 2g,h) showing the distribution of PTFE particles, as discussed in more detail below.

**Figure 9:**
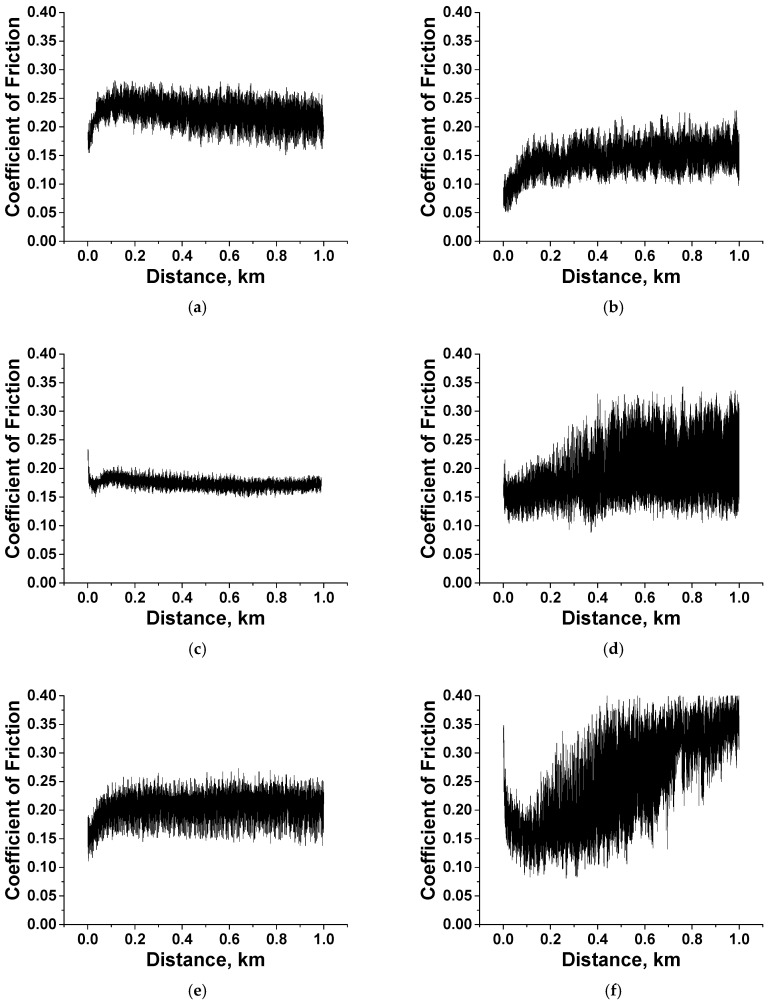

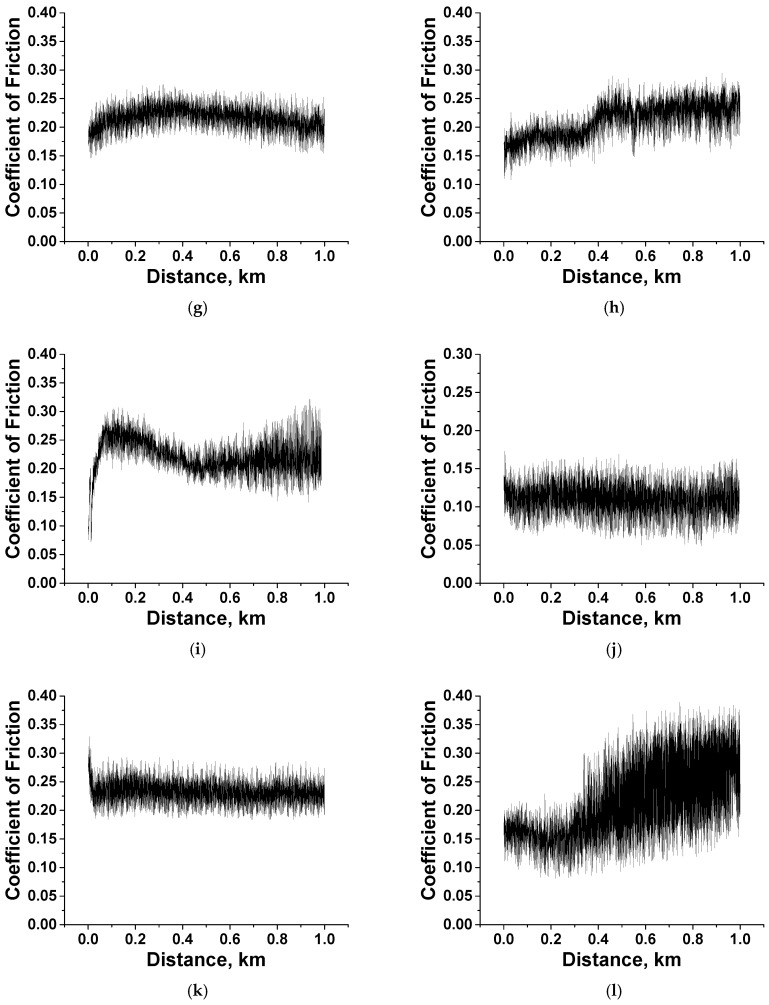
The dependencies vs. distance of the CoF values for the PI/10µPTFE (**a**), PEI/10µPTFE (**b**), PI/10µPTFE/µMoS_2_ (**c**), PEI/10µPTFE/µMoS_2_ (**d**), PI/10µPTFE/µGr (**e**), PEI/10µPTFE/µGr (**f**), PI/5nanoPTFE (**g**), PEI/5nanoPTFE (**h**), PI/5nanoPTFE/µMoS_2_ (**i**), PEI/5nanoPTFE/µMoS_2_ (**j**), PI/5nanoPTFE/µGr (**k**), and PEI/5nanoPTFE/µGr (**l**) composites under the “B-o-R” scheme; *p* = 60 N, *V* = 0.3 m/s.

Figure 9 shows time dependences of the CoF values for the PEI- and PI-based composites at *p* = 60 N. For the PI-based composites, they remained at an almost constant level (corresponding to their mean values, according to Table S3) at the end of the run-in stage (typically not exceeding 100 m). For the PI/10µPTFE/0.5µMoS_2_ sample (Figure 9c), the CoF–distance dependence was characterized by low-level, high-frequency (HF) oscillation (its range was less than 0.03). This fact could indicate that a tribofilm formed and adhered on the wear track surface; however, additional investigation/confirmation is required. In all other cases (including the two-component nanocomposite), the HF–oscillation range was above 0.05 (Figure 9e,g,i), while the WR values were low and comparable (with a certain exception of PI/5nanoPTFE/0.5µMoS_2_ with WR = 6.3 × 10^−6^ mm^3^/N·m).

For the PEI composites, unstable CoF variations were observed, which were simultaneously accompanied by high-level, high-frequency oscillation (for ones containing MoS_2_, Figure 9d,f,j). We consider that this fact was associated with MoS_2_ (and partly Gr) oxidative activity, which, together with the presence of oxygen atoms in PEI, contributed to the adherence of debris on the wear track surface, complicating the sliding of such composites against the steel counterpart. For the PEI/nanoPTFE composite, the lower HF–oscillation level corresponded to the highest WR value of 51.80 × 10^−6^ mm^3^/N m. In this case, large inclusions of coalesced PTFE in the amount of 5 wt.% did not result in the formation of any tribofilm. A similar effect was evident for the three-component PEI/5nanoPTFE/0.5µMoS_2_, when CoF was unprecedentedly low at 0.11, while the WR exceeded one for the neat PEI (WR = 20.9 and 14.6 × 10^−6^ mm^3^/N·m, respectively).

Thus, all PI-based composites were characterized by lower WR values of (2.11–2.89) 10^−6^ mm^3^/N·m at the low range of the CoF levels of 0.173–0.233. For the PEI-based composites, their WR values differed by more than an order of magnitude at similar CoF levels.

**Figure 10:**
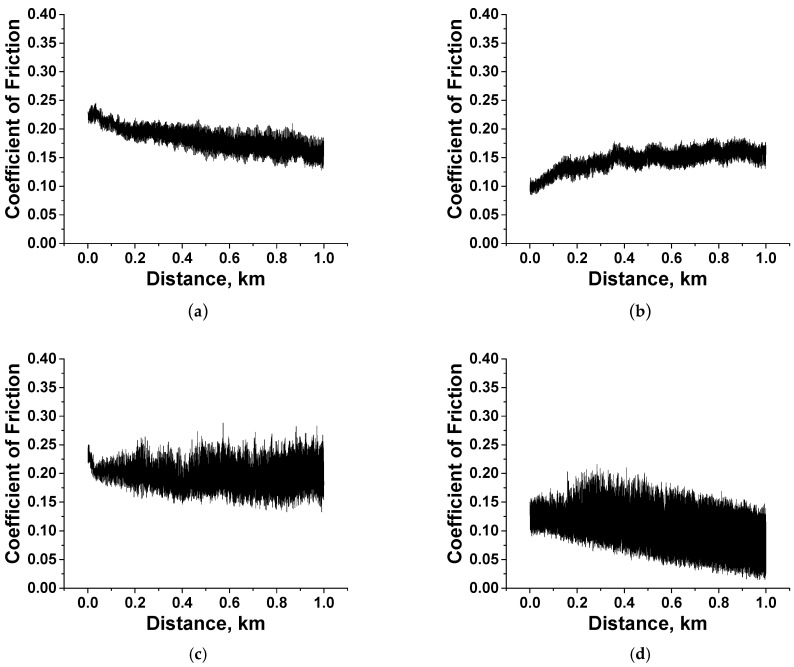

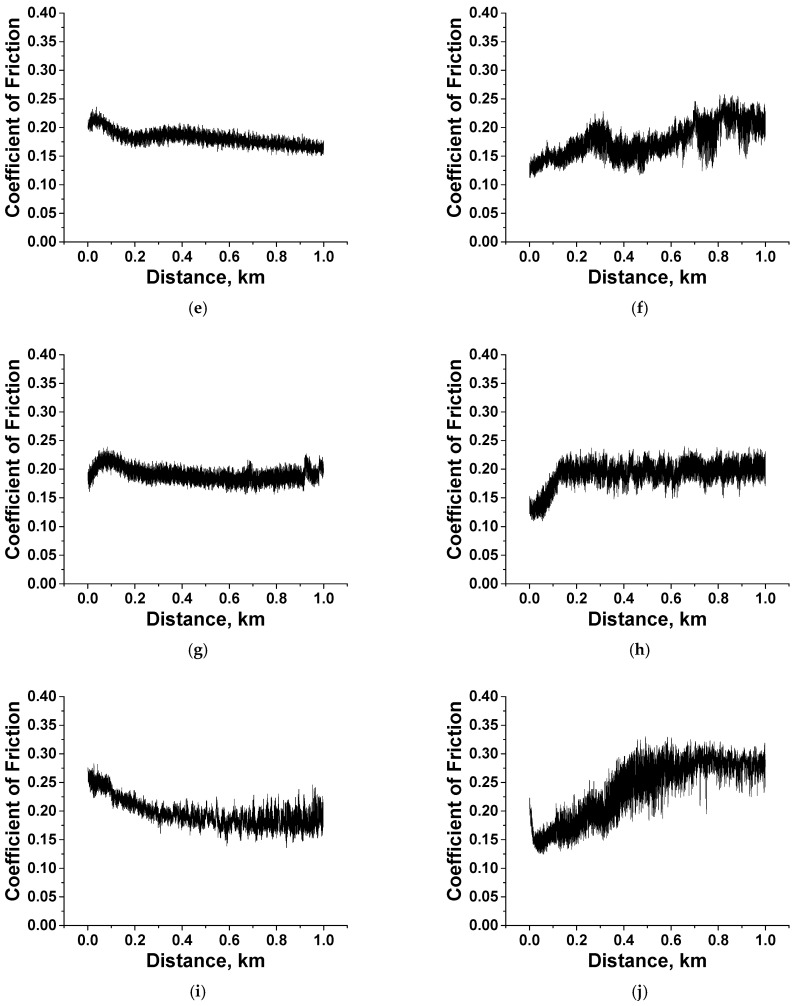

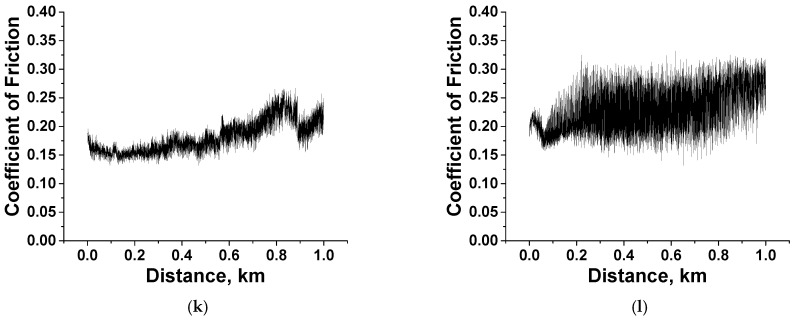
The dependencies vs. distance of the CoF values for the PI/10µPTFE (**a**), PEI/10µPTFE (**b**), PI/10µPTFE/µMoS_2_ (**c**), PEI/10µPTFE/µMoS_2_ (**d**), PI/10µPTFE/µGr (**e**), PEI/10µPTFE/µGr (**f**), PI/5nanoPTFE (**g**), PEI/5nanoPTFE (**h**), PI/5nanoPTFE/µMoS_2_ (**i**), PEI/5nanoPTFE/µMoS_2_ (**j**), PI/5nanoPTFE/µGr (**k**), and PEI/5nanoPTFE/µGr (**l**) composites under the “B-o-R” scheme; *p* = 180 N, *V* = 0.3 m/s.

Figure 10 shows identical CoF–distance dependences for the same composites at the load of *p* = 180 N. In terms of the nature of the CoF oscillations, the pattern changed for the PI-based composites. For the PI/10µPTFE/0.5µMoS_2_ composite, the HF–oscillation level increased with an increase in the testing distance (Figure 10c), although its WR value was very low (1.32 × 10^−6^ mm^3^/N·m). The same WR value was observed for the PI/10µPTFE/0.5µGr composite at a low-level HF oscillation of the CoF–time dependence (Figure 10e). For the two- and three-component composites loaded with nanoPTFE, identical CoF levels were accompanied by a four-fold difference in their WR values (Figure 10g,I,k). It should be noted that the WR value was low for the three-component nanocomposite (i.e., PEI/5nanoPTFE/0.5µGr), despite its PTFE content being only 5 wt.%. This fact indicated that the polymer matrix type and the pattern in the distribution of solid lubricant inclusions significantly affected the tribological characteristics.

In the case of the three-component PEI-based micro-composites, the CoF levels did not show any constant trend with increases in the testing distance (Figure 10d,f,h). As for the PEI/nanoPTFE two- and three-component micro-composites, the WR values were very high (27.1–27.8 × 10^−6^ mm^3^/N·m, see Table S3) at unstable low CoF levels (0.15–0.25, Figure 10h,j,l).

Thus, a clear correlation between decreasing CoF and increasing WR values was not observed for the studied composites during linear tribological contact. In contrast to the three-component PEI-based composites, the WR values were consistently low at 1.32–1.87 10^–6^ mm^3^/N·m (Table S3) for all three PI-based analogs, while the CoF levels were mostly constant during the tribological tests (Figure 10g,I,k). This fact supported the thesis that the formation of an antifriction tribofilm on the wear track surfaces should be the cause.

**Figure 11:**
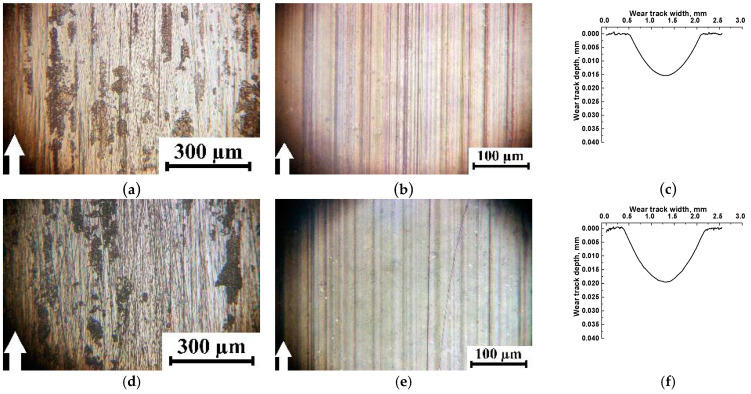

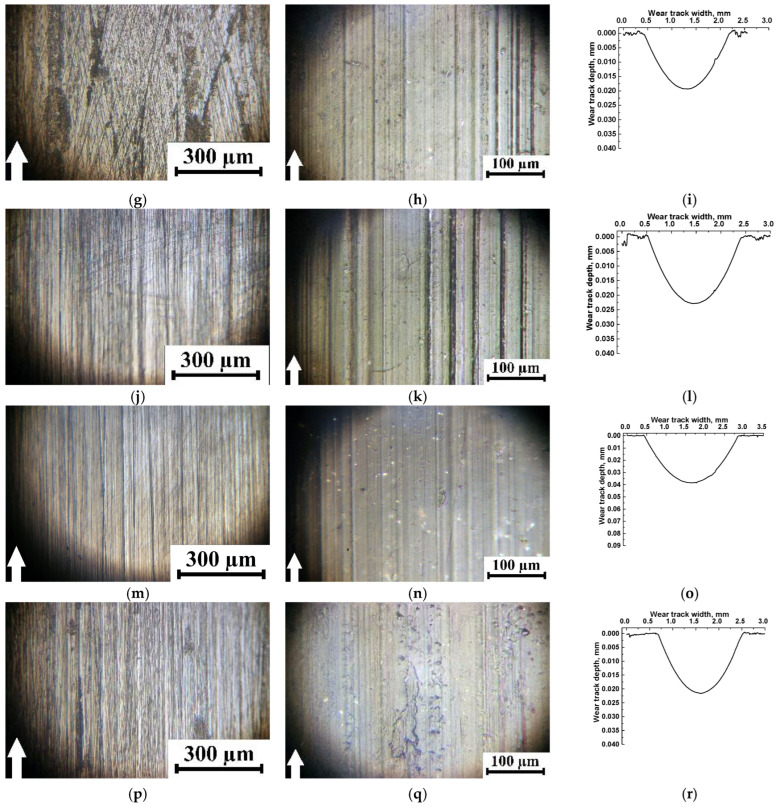
Optical photographs showing the steel counterface (**a**,**d**,**g**,**j**,**m**,**p**), wear track (**b**,**e**,**h**,**k**,**n**,**q**) surfaces, and wear track profiles (**c**,**f**,**i**,**l**,**o**,**r**) after tribological tests of the PI/10µPTFE (**a**–**c**), PI/10µPTFE/µMoS_2_ (**d**–**f**), PI/10µPTFE/µGr (**g**–**i**), PI/5nanoPTFE (**j**–**l**), PI/5nanoPTFE/µMoS_2_ (**m**–**o**), and PI/5nanoPTFE/µGr (**p**–**r**) composites under the “B-o-R” scheme; *p* = 60 N, *V* = 0.3 m/s.

**Figure 12:**
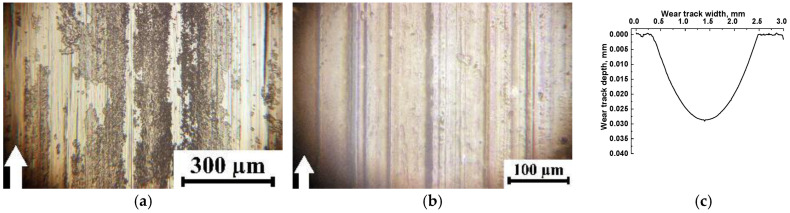

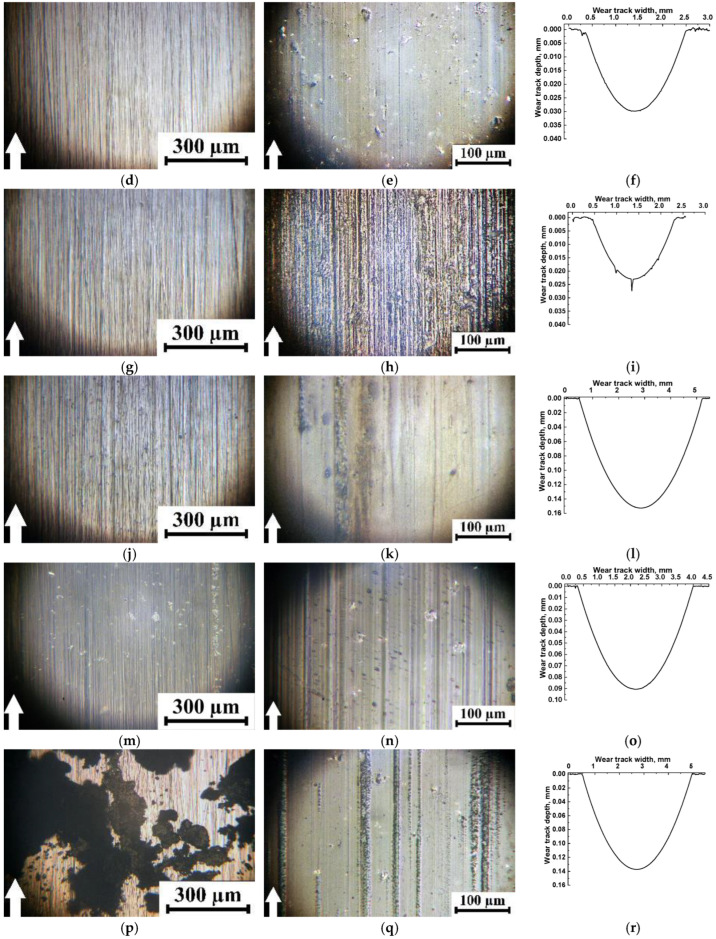
Optical photographs showing the steel counterface (**a**,**d**,**g**,**j**,**m**,**p**),wear track (**b**,**e**,**h**,**k**,**n**,**q**) surfaces, and wear track profiles (**c**,**f**,**i**,**l**,**o**,**r**) after tribological tests of the PEI/10µPTFE (**a**–**c**), PEI/10µPTFE/µMoS_2_ (**d**–**f**), PEI/10µPTFE/µGr (**g**–**i**), PEI/5nanoPTFE (**j**–**l**), PEI/5nanoPTFE/µMoS_2_ (**m**–**o**), and PEI/5nanoPTFE/µGr (**p**–**r**) composites under the “B-o-R” scheme; *p* = 60 N, *V* = 0.3 m/s.

Before discussing the results of this study, we considered it necessary to describe the morphology of the wear track surfaces on the studied composites (Figure 11 and Figure 12).

On the wear track surfaces of the PI-based composites (Figure 11b,e h,k,n,q), shallow longitudinal microgrooves were formed (indistinguishable on their profilograms (Figure 11c,f,i,l,r), since stylus scanning was performed in parallel). At the same time, fragments of the adhered irregularly shaped debris were observed on the steel counterface (Figure 11a,d,g), but this was not observed after the tribological tests of the nanocomposites (Figure 11j,m,p). It should be noted that some of the adhered debris fragments were found on the wear track surface of the three-component nanocomposite (Figure 11q). We consider that the presence of the longitudinal grooves indicated a lower adherence ability for the PTFE-containing tribofilm, although the WR values decreased by more than an order of magnitude compared to that for neat PI.

A similar pattern was observed for the PEI-based composites (Figure 12). The only difference was that the wear track depths were an order of magnitude greater on the PEI/5nanoPTFE (Figure 12j–l), PEI/nanoPTFE/µMoS_2_ (Figure 12m–o), and PEI/nnoPTFE/µGr composites (Figure 12p–r) than those on their PI-based analogs. Most likely, this was caused by an insufficient amount of initial nanoPTFE particles coalesced into large inclusions (up to 30 µm) in the PEI matrix (Figure 2h–j).

## 4. Discussion

The main factors determining the tribological characteristics of the studied composites were (i) the differences in the polymer matrix materials (they ensured the distribution patterns and the sizes of PTFE particles) and (ii) the tribological contact scheme (in terms of both roughness and area of the counterparts). The obtained results are discussed below in this way. Nevertheless, the issue regarding the formation and adherence of the PTFE-containing tribofilms on both friction surfaces was analyzed as the key factor. Note that the aspects of the formation and adherence of both tribofilms and TFs are also considered a priority in the literature on PI/PEI composites [13].

The difference between the problem statement in this paper and the literature data is as follows. Loading with nanoparticles does not fundamentally change the tribological properties, but it could contribute to their substantial increase. The effect is based on the ability of nanoparticles to oxidize and adhere to the transfer film [23]. At the same time, the presence of CNTs ensures the formation of a smoother and more durable wear track surface [22]. In this paper, much like the literature method for introducing MoS_2_ nanoflowers into PI, it was found that adherence to the transfer film would be intensified due to the oxidation ability of molybdenum disulfide. Note, the WR was reduced by 73% in [22].

In addition, the literature describes a positive effect of filling with graphene on increasing the wear resistance of PI-CNT composites, while the former was additionally activated by fluorination [25]. Note that fluorinated graphene (FG) provides an antifriction property. A similar effect was observed with the introduction of nanoCuO in [26]. However, the obtained result of changes in tribological performance significantly depended on the testing conditions [26,27], which include the tribological contact scheme, the roughness of the counterface, load-speed product, etc.

**Figure 13:**
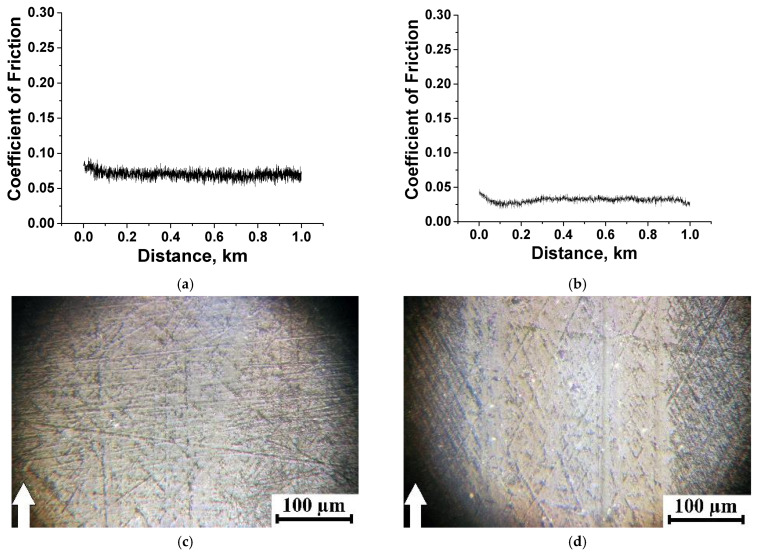
The dependencies vs. distance of the CoF values for the PI/10µPTFE/µMoS_2_ (**a**) and PEI/10µPTFE/µMoS_2_ (**b**) composites and their wear track surfaces (**c**,**d**) under the “B-o-D” scheme; *p* = 5 N, *V* = 0.3 m/s, the ceramic counterpart.

For the point tribological contact, a commercial steel bearing ball with an extremely low *R_a_* = 0.02 µm was utilized. Despite its reactivity, it was not an easy task to adhere a PTFE-containing TF to this steel bearing ball. For comparison, a ZrO_2_ ceramic ball with the same *R_a_* roughness was also used to evaluate the effect of the counterface activity (Table 4; data for the steel ball are reused from the text above). In terms of both parameters, a change in the counterpart material did not exert a significant effect. Thus, a tribofilm formed primarily on the wear track, but the smooth ceramic surface did not remove it (Figure 13c,d). The process was accompanied by an extremely low WR value. Distance dependencies of the CoF values are shown in Figure 13a,b.

For the linear tribological contact, the combination of factors changed: (a) the roughness was higher at *R_a_* = 0.2 µm; (b) there was not enough PTFE to form a tribofilm/TF; and (c) the large and periodically “renewed” surface of the counterpart did not allow the adherence of a protective PTFE-containing tribofilm even with the additional loading with µMoS_2_/µGr. Such conditions were more relevant for the tribological tests of the PEI-based composites, the matrix material of which was more chemically active. So, the question arose: is it possible to reduce the CoF levels down to ~0.1 with a multiple decrease in WR values below WR = 1 × 10^−6^ mm^3^/N m?

**Figure 14:**
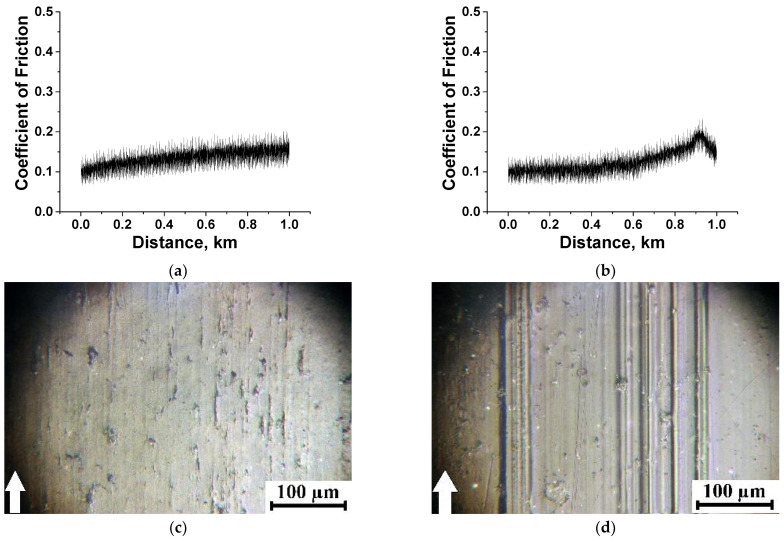
The time dependencies of the CoF values for the PI/10µPTFE/µMoS_2_ (**a**) ad PEI/10µPTFE/µMoS_2_ (**b**) composites and their wear track surfaces (**c**,**d**) under the “B-o-D” scheme; *p* = 5 N, *V* = 0.3 m/s; the ceramic counterpart, *p* = 60 N, *V* = 0.3 m/s under the “B-o-R” scheme.

The answer to this question is partly given in Table 5 and Figure 14, which present the results of the tribological tests against the ceramic counterpart (the data on the steel counterpart are duplicated from those described above). For the three-component micro-composites, the CoF levels reduced to ~0.13, close to achieving the “antifriction” status. For the PI-based micro-composites, it was possible to realize a friction mode almost close to the “wear-less” type (WR = 0.1 × 10^−6^ mm^3^/N m), which was not achieved even during point tribological contact. At the same time, the WR value of 1.3 × 10^−6^ mm^3^/N m was at least an order of magnitude higher for the PEI-based composite.

For the PI-based composites, the wear track surfaces were smooth without any evident microgrooves (Figure 14c), which was characteristic of the counterpart surface. For the PEI-based composites, wear was accompanied by the formation of deep grooves oriented along the sliding direction (Figure 14d). Most likely, this result was related to the less uniform distribution of µPTFE particles in the PEI matrix (Figure 2f), additionally containing 0.5% MoS_2_, and its greater reactivity. The latter could contribute to the debris adhering on the wear track surface and its subsequent tearing out. As a result, the CoF level gradually increased with increasing testing distance (Figure 14b), which was not observed for the three-component PI-based micro-composite (Figure 14a).

Additionally, in terms of achieving the maximum wear resistance for the PI- and PEI-based composites in the linear tribological contact, the results of the tribological tests for three-component composites reinforced with CCFs [32] are given in Table 5. For the steel counterpart, it was possible to obtain low WR values of (1.13–1.39) × 10^−6^ mm^3^/N m for the PEI- and PI-based composites, respectively. At the same time, their CoF levels were 0.18 and 0.24, respectively (which were comparable with the results of this study). However, the mechanism for achieving the maximum wear resistance was determined with the formation of a tribological layer [32], namely, the matrix polymer mixing layer reinforced with partially fractured CCFs. The presence of PTFE in the three-component composites loaded with CCFs did not allow for the formation a tribolayer, so the WR values increased up to (14.6–26.8) × 10^−6^ mm^3^/N m for the PEI- and PI-based composites, respectively (Table 5).

In this study, the absence of CCFs in the composites ruled out the formation of a tribological layer, but this was compensated by the presence of the PTFE-containing composites. This made it possible to reduce the CoF levels by ~2 times compared with that for neat PI and PEI, but it did not exclude wearing of the composites. A minimum WR of 2.63 × 10^−6^ mm^3^/N m was observed for the PI/10µPTFE/0.5µMoS_2_ sample tested against the steel counterpart (a WR of 2.40 × 10^−6^ mm^3^/N m was registered for the PI/10µPTFE/0.5µGr sample).

Under the “B-o-R” scheme, the question regarding the adhering of a PTFE-containing tribofilm arose separately in light of the chemical inertness of PTFE. Firstly, 0.5 wt.% µMoS_2_ (as well as less active µGr) were purposely loaded to retain it, which could be adhered on both the counterpart and wear track surfaces due to the tendency of MoS_2_ to oxidize. Secondly, the debris contained the primarily (oxidized) polymer matrix material that did not impart antifriction properties. Thirdly, noticeable debris (preferably PTFE-containing) was needed to reduce the steel counterpart roughness by covering it with a continuous TF.

**Figure 15:**
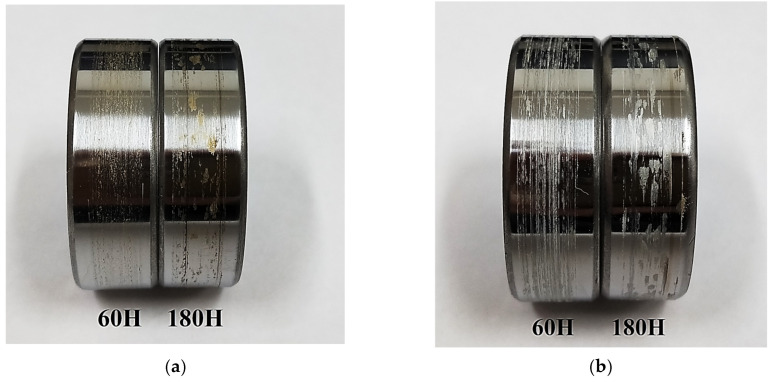
Photographs showing the steel counterpart after tribological tests against the PI/10µPTFE (**a**) and PEI/10µPTFE (**b**) composites under the “B-o-R” scheme; *p* = 60 and 180 N, *V* = 0.3 m/s.

As an illustration, Figure 15 shows photographs of the steel counterpart surfaces after the tribological tests against the PI/10µPTFE and PEI/10µPTFE composites with a threefold difference in the applied loads. Regardless of the matrix material, TFs were formed, which actually represented separated fragments of the matrix material. The color of such fragments corresponded well to those of the matrix in each of the composites. This indicated that: (i) the TFs were not a continuous thin layer; (ii) it was unlikely to obtain a low CoF upon friction, even in the polymer–polymer mode; and (iii) the TF compositions had to correspond to a certain extent to those of the tribofilms on the wear track surfaces.

In this regard, the Raman spectrum of the TF on the steel counterface was analyzed initially under the “B-o-D” scheme. This was motivated by the fact that this TF was solid, and it was of interest to evaluate its composition (including in terms of its ability to adhere on the steel counterpart). Then, the Raman spectrum of the tribofilm was investigated for the PEI-based composites tested according to the “B-o-R” scheme. The tribofilm compositions were compared only for the PEI-based composites since the Raman spectrum peaks were not formed in the PI cases.

**Figure 16:**
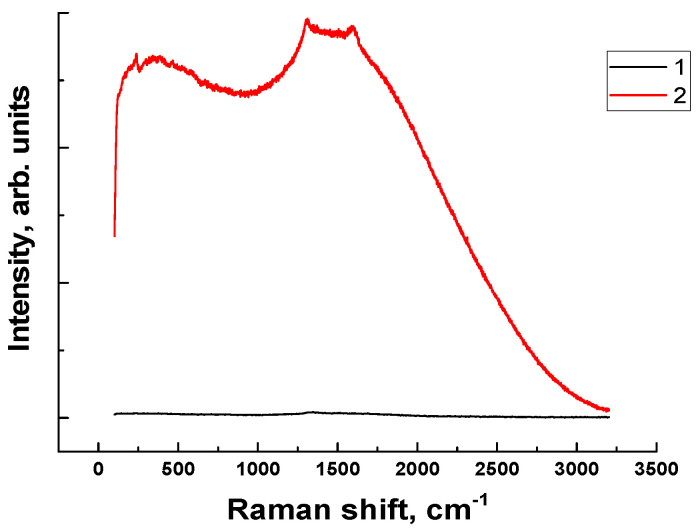
The Raman spectra of the steel counterface: outside (1) and inside (2) the TF after the tribological test of the PEI/10µPTFE/µMoS_2_ composite under the “B-o-D” scheme.

Figure 16 shows Raman spectra registered for the TF on the steel counterpart surface after the tribological tests of the PEI/10µPTFE/µMoS_2_ composite according to the “B-o-D” scheme (curve 2), as well as outside the TF (curve 1). In the latter case, the spectrum was expectedly characterized by zero intensity over the entire range of reciprocal wavelengths (curve 1). On the TF spectrum (curve 2), the intensity increased many times over. In this case, the following characteristic maxima (regions) could be distinguished: (i) in the range of 1000–1670 cm^−1^, the maximum could be attributed to the (C–F, S–F) organofluorine compounds; ii) in the range of 500 cm^−1^ and below, weak peaks were distinguished, which could reflect the (S–O, S–H, C–S) sulfur peaks [33]. These results indicated that the TF contained both PTFE (according to the maximum in the range of 1000–1670 cm^−1^), and the level of this peak was very high. In addition, despite the low content, traces of the more reactive MoS_2_ were detected (according to the appearance of the maximum in the range of 500 cm^−1^ and below). At the same time, the extremely low WR level for this composite, shown in Figure 7e, suggested a minimum PEI content in the TF.

**Figure 17:**
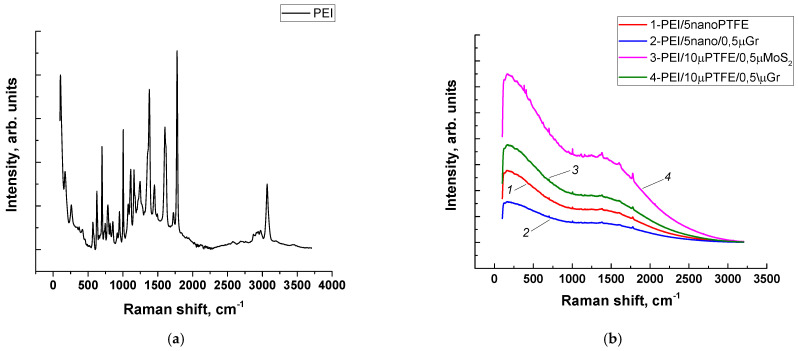
The Raman spectra of the neat PEI (**a**) and the wear track surface of the PEI-based composites after the tribological test (**b**) under the “B-o-R” scheme; *p* = 60, *V* = 0.3 m/s, the steel counterpart.

Figure 17b presents Raman spectra recorded on the wear track surfaces of the PEI-based composites after the tribological tests according to the “B-o-R” scheme. They differed noticeably in intensity. By analogy with the above results, the main maxima were described using the PEI/10µPTFE/µMoS_2_ composite as an example (Figure 17b, curve 4). In the range of 1680–1450 cm^−1^, the peaks belonged to organofluorine compounds: the maximum at the reverse wavelength of 1000 cm^−1^ reflected C–F (valence), 750 cm^−1^ reflected S–F (valence), 1750 cm^−1^ reflected carboxylic acids, and ≤500 cm^−1^ reflected sulfides.

Ranking the spectra in terms of increasing intensity level, the highest intensity was typical for the PEI/10µPTFE/0.5µMoS_2_ composite followed by the analogous PEI/10µPTFE/0.5µGr composites, while the minimum was characteristic of PEI/5nanoPTFE/0.5µGr. At the same time, with the exception of the PEI/5nanoPTFE and PEI/5nanoPTFE/0.5µGr nanocomposites, both three-component PEI-based micro-composites had comparable CoF and WR values. The low level of the maximum in the range of 1680–1450 cm^−1^ likely indicated the insufficient content of (solid-lubricating) PTFE in the tribofilm. However, the combination of the fillers made it possible to obtain its greater uniformity (the absence of microgrooves and a smooth surface in Figure 12e; some similarity is evident for PEI/10µPTFE/0.5µGr, Figure 12h).

For the nanoPTFE two-component composites (with a deficiency of 5 wt.% PTFE and its coalescence), intensive wear was found (Figure 12k), while oxidized debris in the form of thin flakes were fixed on the steel counterpart surface in PEI/5nanoPTFE/0.5µGr (Figure 12p). Nevertheless, the differences in the Raman spectra were not reflected in the WR values. This could additionally indicate that the formation of a stable tribofilm did not occur under such conditions (primarily in the PEI-based composites).

Two more characteristic facts regarding the analysis of Raman spectra should be noted. In the case of the two-component nanocomposite (without MoS_2_/Gr; Figure 17b, curve 1), the spectrum was similar to that for the PEI/5nanoPTFE/0.5µGr nanocomposite but higher in intensity (Figure 17b, curve 2). Thus, the increase in the Raman spectra level in the direction of small reciprocal wavelengths should not be associated with the influence of Gr, but rather, it should be determined by deformation (the formation of a more ordered structure due to the rolling/smoothing effect of the steel counterpart) and, possibly, a tribological fracture of the polymer matrix. The Raman spectrum of neat PEI (in the amorphous state) is shown in Figure 17a. There were a large number of peaks in the spectrum, but their intensity was particularly equal in the entire range of reciprocal wavelengths. Respectively, it was concluded that the wear track of the polymer composite underwent a number of structural changes in the tribological test against the steel counterpart. Such changes were more intense in the case of 10µPTFE and 0.5µMoS_2_/Gr particles.

**Figure 18:**
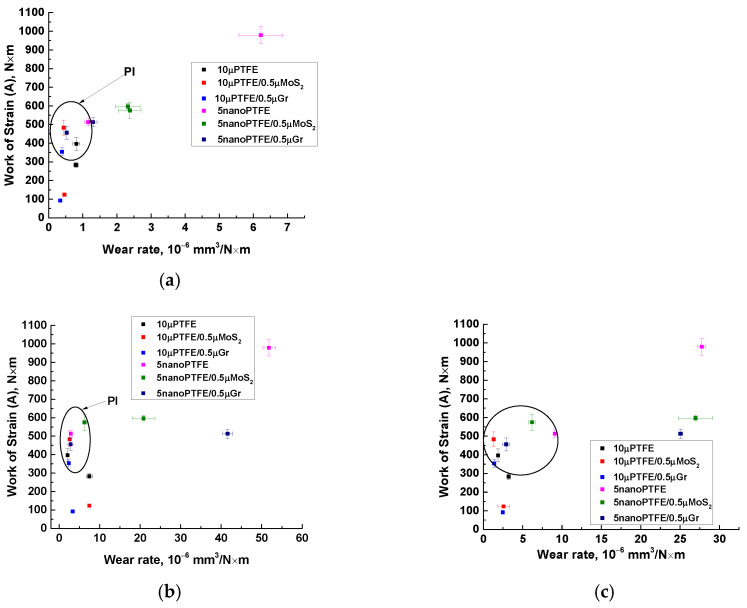
Materials selection approach [34] applied to the qualification of the PI/PEI-based composites for tribological applications; the point (**a**) and linear (**b**,**c**) tribological contact, *p* = 5 N (**a**), 60 N (**b**), and 180 N (**c**); V = 0.3 m/s (**a**–**c**).

Figure 18 shows the combined data on the mechanical and tribological properties of the studied composites, which enable us to recommend a number of them for application in metal–polymer units operating under dry sliding friction conditions. As a characteristic of the mechanical properties, work of strain was applied as the area under the σ–ε curves. This decision was based on the fact that antifriction materials, as a rule, did not possess high strength properties (primarily, the elastic modulus). At the same time, work of strain characterized the ability of the polymer composites to resist applied loads until their failure. In the point tribological contact (Figure 18a), all PI-based composites were grouped in the upper left corner (grouped with a black circle), but the combination of both parameters should give preference to the PI/10µPTFE/µMoS_2_ and PI/10µPTFE/µGr composites.

In the linear tribological contact at the load of 60 N (Figure 18b), all PI-based composites were concentrated in a narrow region, the size of which increased slightly as the load increased up to 180 N (Figure 18c). However, even in this case, we preferred the PI/10µPTFE/µMoS2 composite loaded with the “micro/micro” combination of fillers.

## 5. Conclusions

In the point tribological contacts of the PI- and PEI-based composites additionally loaded with 0.5 wt.% MoS_2_/Gr against the smooth steel counterpart (*Ra* = 0.02 µm), the formation and adherence of both tribofilm and TF contributed to multiple decreases in the WR values due to the realized “antifriction” mode (CoF ≤ 0.1). At the same time, the less uniform distribution of PTFE in the PEI matrix practically did not change the wear resistance of the three-component composites compared to the identically loaded PI-based composites.

During hot pressing, PTFE nanoparticles melted and coalesced into micron-sized porous inclusions. In the PEI matrix, their dimensions were much larger (up to 30 µm) compared to those in the PI matrix (up to 6 µm). This result is of key importance in terms of structure formation. In addition, secondary microfillers tended to agglomerate around them. The phenomenon eliminated PTFE’s role as effective uniformly distributed nanofillers, while the content of 5 wt.% was not always sufficient for the formation of a tribofilm and a significant decrease in the WR values. At the loaded content, the role of MoS_2_/Gr microparticles was similar, although they more successfully solved the problem of adhering a PTFE-containing tribofilm in the point tribological contact. This result is novel in terms of the role of the secondary fillers in providing trobological properties.

In the linear tribological contact, the higher steel counterpart roughness (*Ra* = 0.2 µm), as well as the large area of its sliding surface with the same PTFE content in the three-component PI- and PEI-based composites, did not allow for the formation and adherence of a stable PTFE-containing tribofilm on the wear track surface or a TF on the steel counterpart. As a result, the CoF levels were not below 0.17 for the PI/10µPTFE/0.5µMoS_2_ composite (WR = 2.62 × 10^−6^ mm^3^/N m). For the PEI-based composites, the inability to protect the steel counterpart from contacting the more reactive matrix, especially under the conditions of PTFE deficiency, was accompanied by multiple increases in the WR values, several times greater than those of neat PEI. The unprecedentedly low CoF = 0.11 was revealed for three-component PEI/5nanoPTFE/0.5µMoS_2_; however, it was achieved due to high WR = 20.9 × 10^−6^ mm^3^/N·m (exceeded one for neat PEI) and realization of the polymer-to-polymer rubbing mode.

In the tribological tests according to the “B-o-R” scheme, the more uniform distribution of 10µPTFE in the PI matrix allowed the formation of the protective tribofilm from the very beginning, which provided friction with less HF oscillation of CoF and minimized the WR values.

In the linear tribological contact, the higher activity of the steel counterpart prevented sliding in the antifriction mode (CoF~0.1). The lower activity of the ceramic counterpart, minimizing the possibility of adhering PI-containing debris on the counterpart, provided the multiple lower WR of 0.1 × 10^−6^ mm^3^/N m for the PI/10µPTFE/0.5MoS_2_ composite.

PI/10µPTFE/µMoS_2_ is recommended for application in both point and linear metal-polymer units despite the fact that PI is harder to process into final products.

## Figures and Tables

**Table 1:** The list of powders used for sample fabrication.

Powders	Dimension	Manufacturer
**Matrix**		
PI powder	average particle size of 16 µm	PI-1600, Solver, Jiande, China
PEI powder	average particle size of 20 µm	PEI ROOH, Jiande, China
**Solid lubricants**		
The “Fluralit” fine PTFE powder	average particle size of <3 µm	“Fluralit synthesis” LLC, Moscow, Russia
Molybdenum disulfide	average particle size of 1–7 µm	Climax Molybdenum, Phoenix, AZ, USA
PTFE nanoparticles	average particle size of 100 nm	Daikin, Osaka, Japan
Graphite particles	average particle size of 10 µm	Kegong Metallurgical Materials Co., Ltd., Xingtai, China

**Table 2:** The compositions and designations of the studied composites.

Composition, wt.%	Designation
PI + 10%PTFE (micro)	PI/10µPTFE
PI + 10% PTFE (micro) + 0.5% MoS_2_ (micro)	PI/10µPTFE/0.5µMoS_2_
PI + 10% PTFE (micro) + 0.5% Graphite (micro)	PI/10µPTFE/0.5µGr
PI + 5%PTFE (nano)	PI/5nanoNPTFE
PI + 5%PTFE (nano) + 0.5% MoS_2_ (micro)	PI/5nanoPTFE/0.5µMoS_2_
PI + 5%PTFE (nano) + 0.5% Graphite (micro)	PI/5nanoPTFE/0.5µGr
PEI + 10%PTFE (micro)	PEI/10µPTFE
PEI + 10% PTFE (micro) + 0.5% MoS_2_ (micro)	PEI/10µPTFE/0.5µMoS_2_
PEI + 10% PTFE (micro) + 0.5% Graphite (micro)	PEI/10µPTFE/0.5µGr
PEI + 5%PTFE (nano)	PEI/5nanoNPTFE
PEI + 5%PTFE (nano) + 0.5% MoS_2_ (micro)	PEI/5nanoPTFE/0.5µMoS_2_
PEI + 5%PTFE (nano) + 0.5% Graphite (micro)	PEI/5nanoPTFE/0.5µGr

**Table 3:** The tribological properties of the PI- and PEI-based composites under the “B-o-D” scheme; *p* = 15 N, *V* = 0.3 m/s.

No.	Composite	CoF	WR, mm^3^/N·m, 10^−6^
1	PI/10µPTFE/0.5µMoS_2_/ PEI/10µPTFE/0.5µMoS_2_	0.069 ± 0.002/0.067 ± 0.006	0.54 ± 0.06/0.22 ± 0.02
2	PI/10µPTFE/0.5µGr/ PEI/10µPTFE/0.5µGr	0.069 ± 0.001/0.075 ± 0.004	0.31 ± 0.03/0.20 ± 0.02
3	PI/5nanoPTFE/0.5µMoS_2_/ PEI/5nanoPTFE/0.5µMoS_2_	0.129 ± 0.017/0.137 ± 0.029	1.72 ± 0.16/1.78 ± 0.23
4	PI/5nanoPTFE/0.5µGr/ PEI/5nanoPTFE/0.5µGr	0.114 ± 0.007/0.099 ± 0.003	0.53 ± 0.11/0.38 ± 0.04

**Table 4:** The tribological properties of the PI- and PEI-based composites versus the ceramics\steel counterparts under the “B-o-D” scheme.

No.	Composition (wt.%)	CoF (Steel\Ceramic)	WR, mm^3^/N·m, 10^−6^ (Steel\Ceramic)
1	PI/10µPTFE/0.5µMoS_2_	0.070 ± 0.006\0.085 ± 0.005	0.38 ± 0.04\0.44 ± 0.05/
2	PEI/10µPTFE/0.5µMoS_2_	0.032 ± 0.003\0.053 ± 0.004	0.53 ± 0.06\0.46 ± 0.05

**Table 5:** The tribological characteristics of the PI- and PEI-based composites under the “B-o-R” scheme.

No.	Composite	Load, N	CoF	WR, mm^3^/N·m, 10^–6^	Temperature, °C
Ceramics\Steel counterface					
1	PI/10µPTFE/0.5µMoS_2_	60	0.134 ± 0.019\0.173 ± 0.008	0.10 ± 0.01\2.62 ± 0.27	36.09 ± 1.56\31.6 ± 0.8
2	PEI/10µPTFE/0.5µMoS_2_	60	0.125 ± 0.023\0.211 ± 0.028	1.30 ± 0.15\7.48 ± 0.41	36.81 ± 1.70\25.1 ± 1.1
PI- and PEI composites with carbon fibers/friction against the steel counterface [32]					
3	PI + 10%CCF_2mm_ + 10%PTFE	60	0.318 ± 0.030	26.83 ± 0.90	29.3 ± 0.9/21.3
4	PI + 10%CCF_Mmm_ + 10%Gr	60	0.242 ± 0.023	1.39 ± 0.13	25.1 ± 0.2/21.7
5	PI + 10%CCF_2mm_ + 10%MoS_2_	60	0.267 ± 0.029	1.57 ± 0.42	23.9 ± 0.1/21.4
6	PEI + 10%CCF_2mm_ + 10%PTFE	60	0.206 ± 0.023	14.62 ± 1.14	27.4 ± 0.3/24.6
7	PEI + 10%CCF_2mm_ + 10%Gr	60	0.181 ± 0.016	1.13 ± 0.14	26.8 ± 0.3/24.5
8	PEI + 10%CCF_2mm_ + 10%MoS_2_	60	0.228 ± 0.029	5.15 ± 0.32	28.0 ± 0.3/25.1

## Data Availability

The data presented in this study are available on request from the corresponding author. The data are not publicly available due to confidential disclosure reasons.

## Supplementary Materials

The following supporting information can be downloaded at: https://www.mdpi.com/article/10.3390/polym15153266/s1. Table S1. The physical and mechanical properties of the PI- and PEI-based composites. Table S2. The tribological properties of PI and PEI composites; the “ball-on-disk” scheme. Table S3. The tribological characteristics of the PI- and PEI-based composites; the “block-on-ring” scheme.
