# Supplementary material for: Influence of the Matrix Material and Tribological Contact Type on the Antifriction Properties of Hybrid Reinforced Polyimide-Based Nano- and Microcomposites
Source: Polymers (Basel). 2023 Jul 31;15(15):3266. doi: 10.3390/polym15153266 (PMC10421459; doi:10.3390/polym15153266)
Supplement: Supplementary file 1 [file polymers-15-03266-s001.zip › polymers-2526946-supplementary.pdf]

2.2. Fabrication of the composites

The polymer powders and the fillers were mixed by dispersing the suspension components in alcohol using a "PSB-Gals 1335-05" ultrasonic bath (PSB-Gals Ultrasonic equipment center, Moscow, Russia). The process duration was 5 min at a generator frequency of 22 kHz. After mixing, a suspension of the components was dried in a "Memmert UF110" oven (Memmert GmbH + Co. KG, Schwabach, Germany) at a temperature of 125 °C for 3.5 h. Both PI- and PEI-based nanocomposites were fabricated via hot pressing at a pressure of 15 MPa and a temperature of 370 °C with a subsequent cooling rate of 2 °C/min.

2.3. Physical and Mechanical Properties

Tensile properties of dog-bone shaped samples were measured following ASTM D638 with the use of an "Instron 5582" electromechanical testing machine (Instron, Norwood, MA, USA). The number of samples of each material was at least four. The following samples' gauge dimensions were taken: thickness = 3.2±0.4 mm, width = 3.18±0.5 mm, length = 9.53±0.5 mm. The samples were stretched until their failure.

2.4. Tribological Characteristics

In the point tribological contact according to the "ball-on-disk" scheme, dry sliding friction tests were carried out at a load (*P*) of 5 N and a sliding speed (*V*) of 0.3 m/s. In addition, a triple increased load of 15 N was employed for comparison. A "CH 2000" tribometer (CSEM, Neuchâtel, Switzerland) was used in accordance with ASTM G99. The tribological tests were conducted using a metal counterpart (a ball made of the GCr15 bearing steel, 60 HRC) with a diameter of 6 mm and an R_a surface roughness of ~0.02 μm. The latter parameter was assessed with a "New View 6200" optical interferential profilometer (Zygo Corporation, Middlefield, CT, USA). A testing distance was 1 km and a tribological track radius was 16 mm.

In the linear tribological contact according to the "block-on-ring" scheme, the dry sliding friction tests were performed using a "2070 SMT-1" friction testing machine (Tochpribor Production Association, Ivanovo, Russia). A load (*P*) was 60 N, while a sliding speed (*V*) was 0.3 m/s. In addition, increased both load of 180 N and sliding speed of 0.5 m/s were taken for comparison A metal counterpart was made of the outer ring of a commercial bear (the GCr15 steel, a diameter of 35 mm and a width of 11 mm). Its R_a surface roughness was ~0.20 μm. The number of tested samples of each type was at least four for both types of tribological contacts.

The parameters of the tribological tests were selected by the following reasons: i) the possibility of their comparison with the data from the literature; ii) the possibility of registering the wear parameters for the planed testing time; iii) the duration of the run-in and steady-state wearing stages.

The counterpart temperatures were assessed with a "CEM DT-820" non-contact infrared thermometer (Shenzhen Everbest Machinery Industry Co., Ltd., Shenzhen, China).

WR levels were determined by measuring width and depth of wear tracks according to stylus profilometry (KLA-Tencor, Milpitas, CA, USA), followed by multiplication by their length. They were calculated taking into account both load and distance values:

$$\text{Wear rate} = \frac{\text{volume loss (mm}^3\text{)}}{\text{load (N)} \times \text{sliding distance (m)}} \quad ($$

2.5. Structural Studies

The images of the wear track surfaces were observed using a "Neophot 2" optical microscope (Carl Zeiss, Jena, Germany) equipped with a "Canon EOS 550D" digital camera (Canon Inc., Tokyo, Japan), while wear track profiles were measured with an "Alpha-Step IQ" stylus profiler (KLA-Tencor, Milpitas, CA, USA).

The structural studies were carried out on cleaved surfaces of notched specimens, fractured mechanically after cooling in liquid nitrogen. A "LEO EVO 50" scanning electron microscope (SEM, Carl Zeiss, Oberkochen, Germany) was used at an accelerating voltage of 20 kV.

After the tribological tests, both TFs on the counterpart surface and tribofilms on the wear track surfaces were analyzed using Raman spectroscopy with a "Renishaw inVia Basis Raman" spectrometer (Renishaw plc, Gloucestershire, UK).

Table S1. The physical and mechanical properties of the PI- and PEI-based composites.

No.	Composite	Density ρ, (g/cm^3)	Shore *D* Hardness	Elastic Modulus *E* (GPa)	Ultimate Tensile Strength σ_{UTS} (MPa)	Elongation at Break ε (%)
1	PI/10μPTFE<br>PEI/10μPTFE	1.41/<br>1.31 (93%)	74.6±1.1/<br>75.2±0.3 (100%)	2.54±0.05/<br>2.84±0.02 (119%)	77.7±4.4/<br>84.8±4.5 (109%)	10.3±0.9/<br>6.4±0.5 (62%)
2	PI/10μPTFE/0.5μMoS_2<br>PEI/10μPTFE/0.5μMoS_2	1.42/<br>1.33 (94%)	71.9±0.9/<br>77.0±0.5 (107%)	3.16±0.07/<br>3.49±0.04 (110%)	95.2±0.4/<br>69.8±4.7 (73%)	9.5±0.6/<br>4.9±0.5 (52%)
3	PI/10μPTFE/0.5μGr<br>PEI/10μPTFE/0.5μGr	1.41/<br>1.32 (94%)	72.5±0.4/<br>76.5±0.9 (105%)	3.24±0.14/<br>2.84±0.04 (88%)	90.1±2.8/<br>48.6±4.3 (54%)	7.7±0.8/<br>3.9±0.7 (51%)
4	PI/5nanoPTFE<br>PEI/5nanoPTFE	1.39/<br>1.30 (94%)	71.3±0.4/<br>77.4±0.5 (109%)	2.62±0.09/<br>3.06±0.08 (117%)	98.0±2.4/<br>112.8±0.4 (115%)	10.8±1.1/<br>13.0±2.4 (120%)
5	PI/5nanoPTFE/0.5μMoS_2<br>PEI/5nanoPTFE/0.5μMoS_2	1.41/<br>1.31 (93%)	75.2±0.3/<br>75.3±0.5 (100%)	2.33±0.14/<br>2.62±0,19 (112%)	101.1±5.8 /<br>105.7±7.6 (105%)	9.3±2.2 /<br>8.9±2.4 (96%)
6	PI/5nanoPTFE/0.5μGr<br>PEI/5nanoPTFE/0.5μGr	1.40/<br>1.30 (93%)	73.8±0.2/<br>77.6±0.8 (105%)	3.11±0.15/<br>3.46±0.24 (111%)	102.2±5.6/<br>105.2±1.1 (103%)	8.6±0.7/<br>9.7±0.8 (113%)

Table S2. The tribological properties of PI and PEI composites; the "ball-on-disk" scheme

№	Composition (wt.%)	CoF	WR, mm^3/N·m, 10^{-6}
0	PI/<br>PEI (reference)	0.388±0.012/<br>0.297±0.026	134.16±0.026/<br>390.21±20.51
1	PI/10μPTFE /<br>PEI/10μPTFE	0.098±0.003/<br>0.052±0.003	0.81±0.10/<br>0.8±0.08
2	PI/10μPTFE/0.5μMoS_2/<br>PEI/10μPTFE/0.5μMoS_2	0.085±0.005/<br>0.053±0.004	0.44±0.05/<br>0.46±0.05
3	PI/10μPTFE/0.5μGr/<br>PEI/10μPTFE/0.5μGr	0.107±0.010/<br>0.090±0.004	0.39±0.04/<br>0.34±0.03
4	PI/5nanoPTFE /<br>PEI/5nanoPTFE	0.105±0.012/<br>0.089±0.010	1.16±0.12/<br>6.22±0.64
5	PI/5nanoPTFE/0.5μMoS_2 /<br>PEI/5nanoPTFE/0.5μMoS_2	0.087±0.006/<br>0.152±0.022	2.38±0.33/<br>2.32±0.36
6	PI/5nanoPTFE/0.5μGr /<br>PEI/5nanoPTFE/0.5μGr	0.097±0.013/<br>0.102±0.002	0.53±0.05/<br>1.30±0.12

Table S3. The tribological characteristics of the PI- and PEI-based composites; the "block-on-ring" scheme.

No.	Composite	Load, N	CoF	WR, $mm^3/N \cdot m$, 10^{-6}	Temperature, °C
0	PI/<br>PEI (reference)	60	0.442±0.063/0.379±0.044	20.37±1.03/14.61±1.32	32.9±0.4/32.7±0.4
		180	0.711±0.088/0.463±0.047	39.68±0.82/10.15±0.59	75.1±2.8/51.5±1.4
1	PI/10μPTFE/<br>PEI/10μPTFE	60	0.226±0.024/0.153±0.025	2.11±0.22/7.47±0.76	26.4±0.1/28.4±0.3
		180	0.183±0.017/0.158±0.013	1.87±0.19/3.22±0.17	32.4±0.4/35.5±0.6
2	PI/10μPTFE/0.5μMoS_2/<br>PEI/10μPTFE/0.5μMoS_2	60	0.173±0.008/0.211±0.028	2.62±0.27/7.48±0.41	31.6±0.8/25.1±1.1
		180	0.191±0.011/0.193±0.017	1.32±0.15/2.58±0.74	33.7±1.5/34.9±1.8
3	PI/10μPTFE/0.5μGr/<br>PEI/10μPTFE/0.5μGr	60	0.205±0.022/0.289±0.069	2.40±0.22/3.36±0.19	26.5±0.6/28.1±1.2
		180	0.181±0.011/0.198±0.035	1.39±0.09/2.48±0.23	32.9±1.1/31.3±1.8
4	PI/5nanoPTFE/<br>PEI/5nanoPTFE	60	0.217±0.017/0.191±0.056	2.89±0.32/51.80±1.50	23.3±0.2/26.6±1.1
		180	0.196±0.029/0.147±0.038	9.08±0.07/27.75±0.52	33.2±1.2/31.5±0.8
5	PI/5nanoPTFE/0.5μMoS_2/<br>PEI/5nanoPTFE/0.5μMoS_2	60	0.221±0.029/0.110±0.023	6.30±0.61/20.86±2.82	28.1±1.4/28.4±1.1
		180	0.197±0.025/0.239±0.052	6.19±0.40/26.97±2.17	38.1±2.0/39.2±3.6
6	PI/5nanoPTFE/0.5μGr/<br>PEI/5nanoPTFE/0.5μGr	60	0.233±0.018/0.239±0.062	2.83±0.45/41.60±1.27	25.7±0.9/27.6±1.5
		180	0.182±0.024/0.248±0.039	2.92±0.46/25.07±0.28	32.1±1.1/40.9±2.7
